# The effects of online art therapy on emotional expression and psychological well-being among healthcare professionals and their children during the COVID-19 pandemic

**DOI:** 10.3389/fpubh.2025.1654582

**Published:** 2025-10-13

**Authors:** Muhammet Ali Oruç, Arzu Yalçınkaya, Nurhan Eren

**Affiliations:** ^1^Samsun Universitesi Tip Fakultesi, Canik, Türkiye; ^2^Samsun Il Saglik Mudurlugu, İlkadım, Türkiye; ^3^Istanbul Universitesi Saglik Bilimleri Enstitusu, Fatih, Türkiye

**Keywords:** COVID-19, healthcare professionals, children, art therapy, emotional expression, psychological well-being, psychosocial support

## Abstract

**Objective:**

To explore the effects of an online art therapy intervention on emotional expression and psychological well-being among healthcare professionals and their children aged 9–14 years during the COVID-19 pandemic.

**Methods:**

A single-group pre-post mixed-methods design was employed. The intervention consisted of four online art therapy sessions with healthcare professionals and their children. Quantitative data were collected using validated scales and analyzed with Wilcoxon signed-rank tests. Qualitative data were analyzed with thematic analysis.

**Results:**

The intervention significantly improved emotional expression, psychological well-being, and mental health promotion scores (*p* < 0.05). Qualitative findings indicated enhanced self-awareness, improved family communication, and reduced anxiety.

**Conclusion:**

Online art therapy is a promising psychosocial support strategy for healthcare professionals and their children during crises such as pandemics. Recent studies also highlight the effectiveness of art-based interventions in similar contexts. The importance of such approaches has also been emphasized by creative arts therapists during the COVID-19 pandemic These findings are consistent with recent evidence supporting the role of creative arts therapies for healthcare workers and children. Art therapy interventions delivered online during health crises can play a crucial role in mitigating the psychological burden experienced by healthcare professionals and their families. This study indicates that structured creative interventions improve emotional expression, enhance psychological well-being, and strengthen family communication. Future research should validate these findings with larger samples, longer follow-up, and randomized controlled designs to establish stronger evidence for integrating art therapy into public health preparedness strategies. A quasi-experimental mixed-methods design with a control group was employed. The intervention consisted of four online group art therapy sessions involving healthcare professionals and their children. Quantitative data were collected using the Emotion Expression Scale, Psychological Well-Being Scale, and Mental Health Promotion Scale before and after the intervention. Qualitative insights were gathered through session reflections and participant feedback. Statistical analyses were conducted using the Wilcoxon signed-rank test. The intervention led to a significant improvement in emotional expression, psychological well-being, and mental health promotion scores among participants (*p* < 0.05). Qualitative findings indicated enhanced self-awareness, strengthened family communication, and reduced anxiety among both children and parents. Online art therapy appears to be a promising method for mitigating psychological distress and promoting emotional resilience in healthcare workers and their children during crisis conditions. Integration of creative therapeutic modalities into psychosocial support systems is strongly recommended for future public health emergencies. These findings are consistent with recent evidence supporting the role of creative arts therapies for healthcare workers and children.

## Introduction and objective

In December 2019, a febrile syndrome leading to acute respiratory distress syndrome (ARDS) and lung failure, associated with a previously unknown coronavirus, was reported in Wuhan, China. It began to rapidly spread in the late January 2020. It emerged at the global level as a viral but also a mental pandemic, rapidly putting the world on high alert (1–5).

While all governments struggled with this unexpected COVID-19 pandemic, healthcare professionals worked selflessly with great sacrifice despite intense and stressful working conditions. As a result of these efforts, the healthcare professionals were the group at the highest risk of exposure to COVID-19 infection (6). Healthcare professionals had to cope with challenging situations, including the risk of infection associated with one-to-one contact with patients, physical and mental fatigue caused by intense working conditions, and adapting to the new working system. This had an adverse effect on their mental health as well. Accordingly, the healthcare professionals feared of the risk of transmitting the disease to their relatives (7, 8).

Day-care centers in were closed during the pandemic as a result of restrictive measures in Turkey (9). The restrictions on inter-provincial travel made it impossible for any family member living in other cities to look after the healthcare professionals’ children. The healthcare professionals’ older adults parents were unable to support their children because of the risk of contagion. The child caregivers considered it was inconvenient to care for healthcare professionals’ children and even quit their jobs. Many had to stay at home with their fathers, brothers, sisters, or alone. During this period, the healthcare professionals struggled to find an institution or a person to take care of their children. They could not give a proper hug to their children, when they returned home at the end of their shift. Some left their home away from their children and stayed in a dormitory or hotel. They could not maintain any contact or communication with her children other than video phone calls (9).

The emotion word is derived from the Latin word “emote,” meaning movement. Emotions in action can be most prominently observed in animals and children. Each emotion has a specific effect and a specific role to play. Self-awareness about how one feels gives important clues about the situation being experienced. It provides the opportunity to see the options for future decisions and to change the emotion, if desired to do so (10).

Psychological well-being refers to the activation of one’s potential for self-realization and a meaningful life, when faced with challenges (11). More specifically, psychological well-being is the ability to recognize oneself in positive and realistic terms, to be aware of one’s strengths and limitations, and to act autonomously and independently. Accordingly, it includes finding meaning in life (12).

American Art Therapy Association indicates that this process, which involves artistic self-expression, helps people resolve conflicts and problems, develop interpersonal skills, reduce stress, manage their behavior, increase their self-esteem and self-consciousness, and gain insight thereto. It was reported that creating art with an aim to cope with traumatic experiences can be associated with improvement in people’s cognitive skills and increased enjoyment of life (13, 14).

Art therapy is the method of choice in group therapies and psychoeducation programs, mainly by activating the senses and mediating unconscious processes (15, 16). Besides, Art therapy is a type of therapy that uses creative expression to improve and develop the mental, physical, and emotional health of individuals and can be applied to individuals all age groups, who have different types of issues (17).

This study aimed to investigate the effect of the COVID pandemic on the emotions, expressions, and psychological well-being of parents and children of healthcare professionals with children aged between 9 and 14 years, who worked in healthcare institutions reporting to the Samsun Provincial Health Directorate.

## Materials and methods

### Type of research

This study was conducted as quasi-experimental research with a single-group pre-post design in a mixed design accommodating qualitative and quantitative methods. The researcher developed the application method based on one’s art practice training and literature review. The method development process included supervision of a therapist with experience in art psychotherapy to finalize the practice at the end of the supervision.

### Place and time of research

The study was conducted between 1 July 2020 and 30 March 2021 with healthcare professionals and their children working in health institutions affiliated to Samsun Provincial Health Directorate via Zoom remote interview program.

### Study population and sample

The study population consisted of all the healthcare professionals working in health institutions affiliated to Samsun Provincial Health Directorate and the sample consisted of volunteer healthcare professionals with children aged between 9 and 14 years. Snowball method and announcement via social media platforms were used for the purposes of sample selection in this study group. Using the G-Power Program, the sample size for the intervention group was calculated as 24, considering the intervention group at a moderate level and two-way with an a moderate effect size (Cohen’s d ≈ 0.5).

### Ethical aspects of the research

Required permission was obtained from the Ministry of Health dated 28/04/2020 and numbered 2020–06-17T09_38_34 prior to the commencement of the study. Ethics committee approval dated 20/07/2020 and numbered 2020/11/3 was obtained from the Health Sciences University non-interventional clinical research board. Institutional approval was obtained from the provincial health directorate (dated 20/08/2021 and numbered E-26521195-604.02). Informed consents of the parents and children were collected for the study.

### Qualitative data analysis

Qualitative interviews and session reflections were analyzed using thematic analysis based on Braun and Clarke’s six-step framework (2006). Codes and themes were independently reviewed by two researchers and consensus was reached through discussion. The importance of such approaches has also been emphasized by creative arts therapists during the COVID-19 pandemic (18).

### Data collection tools

#### Child information form

Developed by the researcher. This form consists of items on age, sex, the number of children in the family, whether the child was separated from the family during the COVID-19 pandemic, whether the child was able to adapt to the separation process, and whether the child had physical and mental illness, etc.

#### Family information form

Developed by the researcher. This form consists of items on age, marital status, employment status, educational status, with whom the respondents lived, the time they worked in the pandemic service, whether they were diagnosed with COVID-19 disease, and whether they received institutional support during the pandemic process.

#### Emotion form

This is a list of emotions. A scale between 1 and 10 was introduced to indicate the intensity of emotions. The participants were asked to mark the emotions on the list before the onset of the session and to rate the intensity of that emotion from 1 to 10. At the end of the session, the respondents were asked to write down and rate their emotions at that moment. They were asked whether the emotion they stated prior to the session persisted. Thereafter, they were asked the how they would rate then if the emotion persisted. Participants were asked to rate the session from 1 to 10 on this form. The Wilcoxon test was used to analyze whether there was a significant difference between the mean positive emotion scores of participant healthcare professionals before and after art therapy.

### Emotion expression scale

Developed in 1990 by King and Emmons, this scale aims to measure certain expressions of emotion. The Likert-type scale consists of 16 items, including the tendency to positive, negative, and intimate emotions and each item is scored between 1 and 7 points. The scale items provide information about the interpersonal relationships and emotional expressions of the respondent individuals. Focusing on expressive behaviors, the scale considers both positive and negative emotions (19). The scale was applied before and after art therapy to the participant healthcare professionals. Wilcoxon test was used to analyze whether there was a significant difference between the mean scale scores before and after the session.

#### Psychological wellbeing (PWB) scale

WB was developed by Ryff with an aim to measure psychological wellbeing of university students (20). The 6-point Likert scale consists of 84 items and six factors. Factor scores are obtained along with total scores from the scale. Only the total scores were evaluated for the purposes of the study. The higher the score, the higher the level of psychological wellbeing is considered. The scale was adapted into Turkish language by Cenkseven and Akbaş (21). The total internal consistency coefficient of the Psychological WellBeing Scale was reported as 93. The scale was applied before and after art therapy to the participant healthcare professionals. Wilcoxon test was used to analyze whether there was a significant difference between the mean scale scores before and after the session.

#### Mental health promotion scale

The Mental Health Promotion Scale was conducted as a 47-item Likert-style self-assessment scale (*α* = 0.93). The scale was developed by Kadioglu et al. (16). Scale items There are no reverse scored items included in the scale. The scoring options include Never (1), Rarely (2), Sometimes (3), Frequently (4), Always (5). The minimum and maximum score that can be obtained from the scale is 47 and 23, respectively. Higher scores from the scale are indicative of the individual’s ability to improve his/her mental health increases towards positive direction. The scale has 12 sub-factors (22).

### Application

Participants, who met the inclusion criteria, were briefed about the study and invited to participate. A face-to-face preliminary interview was conducted with the participant, who agreed to participate in the study. During the preliminary interview, detailed information was provided about the purpose of the study, how the data would be used, the content of the study, and possible benefits thereof. The participants were provided with information on the fact that the research would be conducted remotely via Zoom interview, the duration of interview, and the forms to be used. The interview times were scheduled based on the participants’ working hours. Psychological wellbeing scale, emotional expression scale, and mental health promotion scale were administered prior to the first session and following the last session. In the study, each participant was asked to mark which emotion they felt and how intensely they felt it from the list prior to and at the end of the session. At the end of each session, it was assessed with the respondent, whether the emotions and intensity thereof had changed. They were asked what the factors that changed the emotion were and whether the session had an impact thereon. The respondents were then asked to rate on a scale of 1 to 10 the impact of this study on the change in their emotions. Thereafter, they were interviewed about their awareness and thoughts about change.

### Art therapy intervention

With each group consisting of parents and their children, 4 art therapy sessions were performed based on a semi-structured plan according to certain themes. The duration of each session ranged between 60 and 90 min. After a short verbal brief, warm-up, and artwork, emotions, thoughts, and associations about the painting work were collected at the end of the session. Interactions were aimed in this process to ensure self-expression, relaxation, making sense of one’s feelings and raising awareness. Group work was performed online. Participants were informed about the form and procedure of online group work. Audio and video recordings were captured for the purposes of evaluation, reporting and analysis of the group work by experts. The interviewee had participated in the 30-h fall school program focused on “Fundamentals of Art Therapy” organized by Istanbul Life with Art between 5 and 13 October 2019 to acquire required knowledge and competence to use the healing power of art in psychosocial support programs.

1) Session: In the session, participants were asked to provide drawing paper, dry paint, or crayons. The group of parents and children were instructed to “Imagine a place where you can feel safe. Draw it.” This instruction aimed to make the participant develop positive emotions by imagining a place where one could feel happy. How should be the place so the individual can be happy? What are their needs? Ensuring self-awareness. It aims to allow the researcher to get familiar with the participant and establish trust between the participant and the researcher. The participants were asked to describe the place where they felt safe, its characteristics, and how the place made them feel. Questions were asked about the person, objects, and animals drawn in the picture. For instance, if a person drew a tree, whether the tree bore fruit or not, its size, its type. They were asked questions such as what this tree meant to them and how it made them feel.
2) Session. In the session, participants were asked to provide drawing paper, dry paint, or crayons. Participants were instructed, “my mother/father or my child is a hero, he/she can cope with difficulties in difficult situations because of their … traits. Imagine this feature and tell us how you want to draw it, depending on your imagination.” Here, the work especially aimed children under pandemic conditions to realize the strong characteristics of their parents, reduce anxiety, and feel more positive emotions. It was also a study for healthcare professional parents to think about their children’s strengths when they leave them alone at home.
3) Session. In the session, participants were instructed to “Draw a funny cartoon about the COVID-19 virus.” The aim of this session was to imagine the virus in a funny way to reduce its horror and anxiety. Participants shared their thoughts about the virus through the painting. They explained, how they felt by asking questions about their funny thoughts.
4) In the last stage of the art practice session, participants were asked to draw a picture with the instruction “Imagine that the pandemic is over and draw an activity you want to do with your relatives.” The researcher provided the family a single sheet of paper and asked them to draw their dreams about the future on that paper using the collage technique (a painting technique applied by pasting photographs, magazine articles, etc. on a smooth clean surface or mixing them with paint). With this activity, parents and their children used their own drawings along with magazines and colored papers to give shape and form to their own drawings, talking to each other and finalizing the drawing with a joint decision.

## Results

The mean age of the participants was 42.14 ± 5.01, where 83.3% (20) of the participants were female, 91.7% (22) were married, 79.2% (19) were university graduates, and 67% (16) were nurses. The mean years in work of the participants was 20.44 ± 6.07 Min-Max: (9–31) and 62.5% (15) of the participants had no history of mental illness (Table 1).

**Table 1:** Socio-demographic distribution of participant parents.

Variable	*n*	%
Age	42.14 ± 5.01 Min-Max: (34–50)	
Sex		
Female	20	83.3
Male	4	16.7
Marital status		
Married	22	91.7
Divorced	2	8.3
Educational status		
University	19	79.2
Master’s degree	5	20.8
Profession		
Doctor	4	16.7
Nurse	16	66.7
Medical secretary	1	4.2
Dental prosthesis technician	2	8.3
Other	1	4.2
Year in work	20.44 ± 6.07	Min-Max: (9–31)
Socioeconomic status		
Income less than expenditures	5	20.8
Income exceeds expenses	9	37.5
Income equivalent to expenses	10	41.7
Total	24	100
Mental illness status		
No	15	62.5
Yes in the past	7	29.2
Yes ongoing	2	8.3
Total	24	100

54.2% (13) of the participants did not work in the pandemic ward. 12.5% (3) of the healthcare professionals worked voluntarily. 25% (6) of healthcare professionals were diagnosed with COVID, and 20.8% (5) of them had a relative with COVID diagnosis, who died as a result thereof 87.5% (21) stated that they would be able to return to their normal life and work when the pandemic ended (Table 2).

**Table 2:** Participant information form.

Variable	*N*	%
What is your working status in the pandemic service?		
I work voluntarily	3	12.5
I work because it’s may duty	4	16.7
I work compulsorily	4	16.7
I do not work	13	54.2
Have you ever been diagnosed with COVID-19 as a healthcare professional?		
Yes	6	25.0
No	18	75.0
Do you have any family member of relative. Who died due to COVID-19?		
Yes	5	20.8
No	19	79.2
Post-pandemic life		
I can return to my normal life and work. When the pandemic is over		
Yes	21	87.5
No	3	2.5
Total	24	100

66.7% (16) of the participants did not receive financial support from their institutions, 91.7% (22) did not receive support for the family and their children, 95.8% (23) did not receive accommodation and transportation support, and 70.8% (17) did not receive professional knowledge and skills support during the pandemic process. While 37.5% (9) of the participants reported that their working environment was not suitable for their needs, 75% (18) stated that their personal protective equipment was adequate (Table 3).

**Table 3:** Percentage distribution of participants’ institutional support status data.

Type of support	1 = Yes. adequate *n* %		2 = Yes. partially adequate *n* %		3 = No. I did not receive support *n* %	
I received financial support	0	0	8	33.3	16	66.7
I received support for my family and children	0	0	2	8.3	22	91.7
I received support for accommodation and transportation	1	4.2	0	0	23	95.8
I received moral support	1	4.2	1	4.2	22	91.7
I received professional knowledge and skills support	1	4.2	6	25	17	70.8
Was the working arrangement suitable for my needs?	8	33.3	7	29.2	9	37.5
Did you receive the personal protective equipment support you need from your institution during the pandemic?	18	75.0	6	25.0	0	0

The mean age of the participants was 11.11 ± 1.91 Min-Max: (8–15), 66.7% (16) were the first child of the family, 83.3% (20) were not separated during the COVID pandemic, and 8.3% (2) partially adapted to the separation process. 83.3% (20) of the children stayed at home without a caregiver, when their parents went out to work. Participants had no known physical illness. 87.5% (21) had no known psychological disorder (Table 4).

**Table 4:** Participant children information form.

Variable	*n*	%
Age	11.11 ± 1.91 Min-Max: (8–15)	
Sex		
Female	14	58.3
Male	10	41.7
Birth order in the family		
1	16	66.7
2	7	29.2
3	1	4.2
Were your child/children separated from you during COVID?		
Yes	1	4.2
No	20	83.3
Partially	3	12.5
How long did your child live apart from you? …… months		
0.00	20	83.3
1	1	4.2
2	1	4.2
4	1	4.2
6	1	4.2
Was your child able to adapt to the separation process during the pandemic?		
We did not live separately	20	83.3
Yes we had no problem	2	8.3
Partially adapted. Certain sleep disorders and behavior problems	2	8.3
Could you have adapted to being separated from your child?		
We did not live separately	20	83.3
I partially struggled. I wondered about my child/children	4	16.7
Who provided permanent care for your child during your separation?		
At home with siblings (no caregiver)	20	83.3
Father	1	4.2
Relative	3	12.5
Does your child have any previously known physical illnesses?		
No	24	100
Does your child have any previously known mental illness?		
Distraction	1	4.2
Obsessive compulsive disorder	2	8.3
No	21	87.5
Total	24	100.0

The Wilcoxon test was used to analyze whether there was a significant difference between the art therapy pretest and posttest mean scores of the participant healthcare professionals, and the results showed that there was a significant difference (*p* < 0.05) (Table 5).

**Table 5:** A Comparison of art therapy pretest-posttest mean scores of the scales.

Scale/Measure	(*n* = 24) Mean ±	Z score	*p* value
Emotional expression scale pre-session	116 ± 16	−3.184b	0.001
Emotional expression scale post-session	130 ± 22.5		
Psychological wellbeing scale pre-session	130 ± 4	−2.52b	0.012
Psychological wellbeing scale post-session	143 ± 5		
Mental health promotion scale pre-session	95 ± 8.4	−3.21b	0.00
Mental health promotion scale post-session	101.2 ± 9.07		

*p < 0.05. **Wilcoxon test was applied.

The participant healthcare professionals and children were asked about their feelings before and after art therapy and instructed to rate them. Wilcoxon test was used to analyze, whether there was a significant difference between the mean positive scores prior to and after the session. There was a significant difference in the positive emotion scores of the participants in all sessions (*p* < 0.05) (Table 6).

**Table 6:** A comparison of mean positive emotion scores before and after art therapy.

Positive emotion mean scores of children	(*n* = 24) Mean ±	*Z* score	*p* value
Before the 1st session	7.15 ± 2	−4.035	0.00
After the 1st session	8.8 ± 1.4		
Before the 2nd session	7.3 ± 1.6	−4.333	0.00
After the 2nd session	9.07 ± 1.343		
Before the 3rd session	7.56 ± 1.737	−3.888	0.00
After the 3rd session	8.90 ± 1.4		
Before the 4th session	7.04 ± 1.68	−4.143	0.00
After the 4th session	9.01 ± 1.14		
Positive emotion mean scores of parents			
Before the 1st session	6.41 ± 1.5	−11.507b	0.00
After the 1st session	8.32 ± 1.53		
Before the 2nd session	6.54 ± 1.88	−3.850	0.00
After the 2nd session	8.15 ± 0.9		
Before the 3rd session	7.49 ± 2.2	−3.672	0.00
After the 3rd session	8.61 ± 1.1		
Before the 4th session	7.12 ± 1.67	−4.233	0.00
After the 4th session	8.92 ± 1.58		

*p < 0.05. **Wilcoxon test was applied.

The participant healthcare professionals and children were asked to rate the session with a score between (1–10). The mean scores for each session were tabularized (Table 7).

**Table 7:** Mean scores of the participants for the session.

The mean scores given by the children for the sessions	(*n* = 24) Mean ±
Session 1	7.92 ± 1.59
Session 2	8.33 ± 1.47
Session 3	8.25 ± 1.51
Session 4	8.29 ± 1.40

The mean scores given by the parents for the sessions	(*n* = 24) Mean ±
Session 1	7.75 ± 1.42
Session 2	7.67 ± 1.83
Session 3	8.04 ± 1.16
Session 4	8.67 ± 1.13

## Discussion

During an outbreak, healthcare professionals are forced to interact closely with patients for prolonged hours, posing a high risk of contagion. In addition to other mental problems, psychological controversies, especially the risk of secondary trauma, are at a high rate among healthcare professionals, who have to work every day in a setting dominated by epidemic, virus, intensive care, and death (23–25). Healthcare professionals are exposed to higher levels of psychological problems during the pandemic as a result of prolonged working hours, high risk of infection, limited protective equipment, physical fatigue, loneliness, and separation from their families (26). Chinese studies during the COVID-19 pandemic also reported significantly increased anxiety levels in healthcare professionals. It was reported that increased levels of were a factor associated with the high risk of infection, lack of adequate psychological support programs offered by the institution, lack of knowledge about emergency response plans, and high workload (27).

It may prove to be instrumental to schedule group and individual interventions with certain activities, including stress reduction, relaxation techniques, awareness, self-compassion in order to ensure the psychological and physical wellbeing of healthcare professionals, who are at the forefront of the fight against the epidemic (3, 28, 29). Furthermore, it is important to ensure that healthcare professionals hold regular meetings with their families and children, to recognize possible signs of burnout or psychological stress and distress and to carry out proactive prevention activities (30, 31).

In this study, positive (happy, relaxed) and negative (anxiety, sadness, anger) emotions were observed in the emotion-focused sharing and painting study. It was understood from the self-reports of the participants that expression of emotions reduced their anxiety and raised awareness about their inner world. Talking about, understanding, and controlling one’s emotions is considered a part of communication (32). People, who cannot express their own positive and negative emotions are under the threat of a range of mental and organic problems (33). A study, which investigated the preschool children’s aggression levels in terms of emotional expression and emotional regulation, reported that preschool children’s emotional expression skills and emotional expression levels significantly predicted their physical aggression (34).

There were statistically significant results upon assessment of mean positive emotions before and after the session. As a result of a study, which investigated the effect of the Structured Group Art Therapy Program on the happiness levels, tendency to express emotions and emotion regulation difficulties of adolescents between the ages of 15–18, it was reported that there was a significant increase in the positive emotional expression and intimacy emotional expression scores of the sub-factors of the emotional expression tendency scores of the individuals, who participated in a structured art therapy program compared to those who did not (35). A comparison of the results of the above study with the present study indicates similar results.

In this study, art therapy practice had a positive effect on psychological wellbeing. Psychological wellbeing focuses mainly on human development and existential challenges in life (36). A previous study investigated whether mandala practice was effective in increasing the general psychological health, psychological wellbeing and expression of emotions of adult individuals. A pretest-posttest single-group pre-post design experimental design was used for the purposes of the study. As a result, it was reported that there were significant increases in general psychological health, psychological well-being, and expression of emotions scores in the group, which regularly practiced mandala and meditation practice, while there was no significant change in the single-group pre-post design (37). As a result of another experimental study with a single-group pre-post design, which investigated the effect of expressive art therapy on flow state and psychological wellbeing in university students, there was no significant difference between the pre-test and post-test scores in the single-group pre-post design, while there was a significant difference in the experimental group (38). The results of the present study are consistent with the results of both studies above.

In addition to the increased workload created by the pandemic, healthcare professionals also struggle with a series of challenges, including the fear of disease transmission for themselves and their families, new and frequently changing COVID-19 treatment protocols, working with personal protective equipment, caring for large numbers of patients and rapidly deteriorating patients, caring for colleagues who are ill, and deciding on invasive interventions to life support patients. The traumas caused by these difficulties lead to anxiety, fear, frustration, suicidal behaviors, and substance abuse (39). Furthermore, the COVID-19 pandemic led to high rates of severe insomnia, somatization and obsessive-compulsive symptoms in healthcare professionals. The healthcare professionals experienced certain problems, including difficulty falling asleep due to stress, dizziness, irritability, and low mood, impairment in social communication or professional work, and excessive strain from the intensity of daily life (40). The first study with the healthcare professionals in Wuhan, the city where the COVID-19 outbreak was first seen reported that 71.3% of healthcare teams had sub-threshold and mild mental disorders, 22.4% had moderate mental disorders, and 6.2% had severe mental disorders immediately after the outbreak (41). We do not know how the stress experienced by healthcare professionals during the pandemic will affect their future mental health. Although the pandemic lost its severity in Turkey and across world, it is clear that similar dangers await healthcare professionals in the future, associated with many epidemic diseases. It was suggested that expressive therapy methods, including art therapy interventions and psychosocial support aimed to facilitate expressing the stress and emotions might protect mental health.

There are also certain risk factors associated with the effects of COVID-19 on children. The psychology of children and younger people were significantly affected by the family system, and family interactions were deeply shaken by the pandemic. It was suggested that specific concerns about children’s mental health increased the risk of depression, anxiety, or other forms of psychological distress in families (42). This situation is associated with adverse psychological conditions as reflected upon children. This is because children may be more susceptible to the proliferation of stress. As a matter of fact, parents played a crucial role in the COVID-19 process to reduce their children’s stress, to help them manage their emotions, and make sense of their own experiences. Nevertheless, a parent with adequate emotional and physical resources is required to reduce stress. With parents, who increasingly experienced their own demoralizing losses (e.g., loss of jobs, death of loved ones, deterioration of their own mental health, and substance abuse), the ability of parents to reduce their children’s stress inevitably diminished over time, increasing the risk that the pandemic would become more traumatizing and might leave lasting damage on children (43). The art activity practices provided the opportunity to generate a common product and to express feelings mutually through sharing through the work. During the Art Session 2, the participants were instructed, “my child/my mother or my father is a hero, imagine and draw his/her strong traits.” As a result, their paintings described the strong traits of their relatives and their answers to the questions, which deepened each detail, had a positive and supportive effect among family members. Their feedback included such phrases as “I am very happy that my child/parents think this/that about me.”

The participants were instructed to paint the artwork with the collage technique during the last stage of the art practice session. Each participant asked what they meant with a particular figure drawn in the picture. For example, “I brought the sun to warm his love,” “I brought the tree to protect him.” With this practice, working together on the same paper based on various collage and painting materials strengthened the communication between family members through interaction. The activity released the children’s healthcare professional parents from the anxiety of being infected by patients, and the healthcare professional parents from the anxiety of staying away from their children and leaving them alone and made them aware of each other’s strong traits. Analyses in a study, which investigated the effect of art therapy program on individuals’ post-traumatic cognitions, showed that art therapy program had a positive effect on reducing individuals’ post-traumatic cognitions, consistent with the present study (44).

### Limitations

Limitations: (1) the small sample size (*n* = 24), (2) absence of a control group, (3) short-term follow-up, (4) reliance on self-reported measures, and (5) lack of repeated-measures analyses such as the Friedman test due to limited statistical power.

## Conclusion

One of the important results was that the art therapy intervention increased the ability of healthcare professionals to improve their mental health. It was observed in the study that the pessimism caused by the fear and anxiety experienced decreased, and that the desire and efforts to protect and improve mental health increased as expressed in the artistic works and shares.

Emotional expression by healthcare professionals and their children, who were separated from each other during the pandemic, increased through art intervention, and a positive impact on their well-being was achieved. This may ensure protection against further mental problems. We recommend that art therapy interventions should be used as an important method to overcome the adverse effects, especially in unexpected and mass-impact events, including pandemics, and should be included in treatment and support programs.

## Data Availability

The original contributions presented in the study are included in the article/supplementary material, further inquiries can be directed to the corresponding author.

## References

[1] Chen LK. Older adults and COVID-19 pandemic: resilience matters. Arch Gerontol Geriatrics. (2020) 89:104124. doi: 10.1016/j.archger.2020.104124 PMC7247489 32474351

[2] Malboeuf-Hurtubise C Léger-Goodes T Mageau GA Taylor G Herba CM Chadi N. Online art therapy in elementary schools during COVID-19: results from a randomized cluster pilot and feasibility study and impact on mental health. Child Adolesc Psychiatry Ment Health. (2021) 15:15. doi: 10.1186/s13034-021-00367-5, PMID: 33676537 PMC7936482

[3] Engel T Gowda D Sandhu JS Banerjee S. Art interventions to mitigate burnout in health care professionals: a systematic review. Perm J. (2023) 27:184–94. doi: 10.7812/TPP/23.018, PMID: 37303185 PMC10266839

[4] Luo X. Arts therapies for mental disorders in COVID-19 patients. Front Public Health. (2023) 11:1289545. doi: 10.3389/fpubh.2023.1289545 38111478 PMC10726037

[5] Feniger-Schaal R Orkibi H Keisari S Sajnani NL Butler JD. Shifting to tele-creative arts therapies during the COVID-19 pandemic: an international study on helpful and challenging factors. Arts Psychother. (2022) 78:101898. doi: 10.1016/j.aip.2022.101898, PMID: 35221415 PMC8860746

[6] Rajkumar RP. Ayurveda and COVID-19: where psychoneuroimmunology and the meaning response meet. Brain Behav Immun. (2020) 87:8–9. doi: 10.1016/j.bbi.2020.04.056, PMID: 32334064 PMC7175849

[7] Genç BN. Critical management of COVID-19 pandemic in Turkey. Front Life Sci Related Technol. (2020) 1:69–73.

[8] Teksin G Uluyol ÖB Onur ÖS Teksin MG Ozdemir HM. Stigma-related factors and their effects on health-care workers during COVID-19 pandemics in Turkey: a multicenter study. SiSli Etfal Hastanesi Tip Bulteni. (2020) 54:281–90. doi: 10.14744/SEMB.2020.02800, PMID: 33312024 PMC7729714

[9] Bakanlığı Tİ. Koronavirüs ile mücadele kapsamında-yeni kısıtlama ve tedbirler genelgeleri. Çevrimiçi. (2020). Available online at: https://www.icisleri.gov.tr/koronavirus-ile-mucadele-kapsaminda-sokaga-cikma-kisitlamalari---yeni-kisitlama-ve-tedbirler-genelgeleri-html (Accessed March 15, 2021).

[10] Barrett L Gross J Conner T Benvenuto M. Knowing what you’re feeling and knowing what to do about it: mapping the relation between emotion differentiation and emotion regulation. Cogn Emot. (2001) 15:713–24. doi: 10.1080/02699930143000239

[11] Ryff CD Singer BH. Know thyself and become what you are: a eudaimonic approach to psychological well-being. J Happiness Stud. (2008) 9:13–39. doi: 10.1007/s10902-006-9019-0

[12] Keyes CLM Ryff CD. Chapter 7 - psychological well-being in midlife In: Willis SL Reid JD, editors. Life in the middle. San Diego: Academic Press (1999). 161–80.

[13] American Art Therapy Association, About Art Therapy. (2009). Available online at: http://www.arttherapy.org/aboutart.htm (Accessed March 10, 2024).

[14] Moula Z. A systematic review of arts-based interventions delivered in nature and their impact on children’s well-being. Front Psychol. (2022) 13:858781. doi: 10.3389/fpsyg.2022.858781 35350736 PMC8957942

[15] Malchiodi CA. Art therapy and the brain. Handbook of art therapy. New York, NY: Guilford Press. (2003). p. 16–24.

[16] Rubin DB. Using propensity scores to help design observational studies: application to the tobacco litigation. Health Serv Outcome Res Methodol. (2001) 2:169–88. doi: 10.1023/A:1020363010465

[17] Heckwolf JI Bergland MC Mouratidis M. Coordinating principles of art therapy and DBT. Arts Psychother. (2014) 41:329–35. doi: 10.1016/j.aip.2014.03.006

[18] Keisari S Biancalani G Tavelli E Fassina S Testoni I. Spirituality during COVID-19 in northern Italy: the experience of participating in an online prayer group. Pastoral Psychol. (2022) 71:201–15. doi: 10.1007/s11089-022-00998-1, PMID: 35291711 PMC8915138

[19] Kuzucu Y. Duyguları İfade Etme Ölçeği’nin Uyarlanması: Geçerlik Ve Güvenirlik Çalışmaları. Kastamonu Eğitim Dergisi. (2011) 19:779–92.

[20] Ryff CD. Happiness is everything, or is it? Explorations on the meaning of psychological well-being. J Pers Soc Psychol. (1989) 57:1069–81. doi: 10.1037/0022-3514.57.6.1069

[21] Cenkseven F Akbaş T. Üniversite Öğrencilerinde Öznel ve Psikolojik İyi Olmanın Yordayıcılarının İncelenmesi. Turk Psychol Couns Guid J. (2007) 3:43–65. doi: 10.17066/pdrd.57116

[22] Kadioglu H Karaca S Eren N Yurt S. Development and validation of the mental health promotion scale. Perspect Psychiatr Care. (2022) 58:229–38. doi: 10.1111/ppc.12925, PMID: 34374092

[23] Cetintepe SP İlhan MN. COVİD-19 Salgınında Sağlık Çalışanlarında Risk Azaltılması. J Biotechnol Strateg Health Res. (2020) 4:50–4. doi: 10.34084/bshr.712539

[24] Chen Q Liang M Li Y Guo J Fei D Wang L. Mental health care for medical staff in China during the COVID-19 outbreak. Lancet Psychiatry. (2020) 7:e15–6. doi: 10.1016/S2215-0366(20)30078-X, PMID: 32085839 PMC7129426

[25] Holmes EA O’Connor RC Perry VH Tracey I Wessely S Arseneault L. Multidisciplinary research priorities for the COVID-19 pandemic: a call for action for mental health science. Lancet Psychiatry. (2020) 7:547–60. doi: 10.1016/S2215-0366(20)30168-1, PMID: 32304649 PMC7159850

[26] Kang L Li Y Hu S Chen M Yang C Yang BX. The mental health of medical workers in Wuhan, China dealing with the 2019 novel coronavirus. Lancet Psychiatry. (2020) 7:e14. doi: 10.1016/S2215-0366(20)30047-X, PMID: 32035030 PMC7129673

[27] Pan R Zhang L Pan J. The anxiety status of Chinese medical workers during the epidemic of COVID-19: a meta-analysis. Psychiatry Investig. (2020) 17:475–80. doi: 10.30773/pi.2020.0127, PMID: 32403209 PMC7265026

[28] Mukhtar S. Mental health and psychosocial aspects of coronavirus outbreak in Pakistan: psychological intervention for public mental health crisis. Asian J Psychiatr. (2020) 51:102069. doi: 10.1016/j.ajp.2020.102069, PMID: 32344331 PMC7161472

[29] Polizzi C Lynn SJ Perry A. Stress and coping in the time of COVID-19: Pathways to resilience and recovery. Clin Neuropsychiatry. (2020) 17:59. doi: 10.36131/CN20200204 34908968 PMC8629051

[30] Cyrus S. H. H. Cornelia Y. I. C. ve Roger C. M. H.. (2020). Mental health strategies to combat the psychological impact of COVID-19 beyond paranoia and panic, Acad Med, 49, 155–160. 32200399

[31] Enli Tuncay F Koyuncu E Özel Ş. Pandemilerde Sağlık Çalışanlarının Psikososyal Sağlığını Etkileyen Koruyucu ve Risk Faktörlerine İlişkin Bir Derleme. Ankara Med J. (2020) 2:488–501. doi: 10.5505/amj.2020.02418

[32] Ergin FE Üniversite öğrencilerinin sahip oldukları duygusal zeka düzeyi ile 16 kişilik özelliği arasındaki ilişki üzerine bir araştırma: Sosyal Bilimler Enstitüsü (2000)

[33] Yavuzer N. İletişim ve etkili yaşam kültürü, çocuklarımız için eğitim sohbetleri. Ankara: Pegem A Yayıncılık (2000).

[34] Ersan C Tok Ş. Okul öncesi dönem çocuklarının saldırganlık düzeylerinin duygu ifade etme ve duygu düzenleme açısından incelenmesi. Eğitim ve Bilim. (2019) 45

[35] Karataş E Güler ÇY. Grup sanat terapisi programının ergenlerin mutluluk düzeyleri, duyguları ifade etme eğilimi ve duygu düzenleme güçlüğüne etkisi. OPUS Int J Soc Res. (2020) 15:3328–59.

[36] Keyes CL. The mental health continuum: from languishing to flourishing in life. J Health Soc Behav. (2002) 43:207–22. doi: 10.2307/3090197, PMID: 12096700

[37] Ümmet D. Yetişkin bireylerle yürütülen mandala uygulamalarının genel psikolojik sağlık, psikolojik iyi oluş ve duyguları ifade etme becerisi üzerindeki etkisi. (2023).

[38] Kaya A. Dışavurumcu sanat terapinin üniversite öğrencilerinde akış durumu ve psikolojik iyi oluş üzerindeki etkisi (Yüksek lisans tezi). İstanbul: Üsküdar Üniversitesi (2014).

[39] Tanrıverdi Ö Tanrıverdi S. COVID-19’un sağlık çalışanlarının ruh sağlığına etkisi ve ruhsal travmaların önlenmesi. Sağlık Akademisyenleri Dergisi. (2021) 8:245–8.

[40] Fava GA McEwen BS Guidi J Gostoli S Offidani E Sonino N. Clinical characterization of allostatic overload. Psychoneuroendocrinology. (2019) 108:94–101. doi: 10.1016/j.psyneuen.2019.05.028, PMID: 31252304

[41] İzci F. COVID-19 salgını ve sağlık çalışanları (COVID-19 pandemy and health workers). Anatolian J Psychiatry. (2020) 21:335.

[42] Bitsika V Sharpley CF Andronicos NM Agnew LL. Prevalence, structure and correlates of anxiety-depression in boys with an autism spectrum disorder. Res Dev Disabil. (2016) 49-50:302–11. doi: 10.1016/j.ridd.2015.11.011, PMID: 26771668

[43] Çilhoroz Y. COVID-19 Pandemisinin Çocuklar Üzerindeki Psikolojik Etkilerinin İncelenmesi. Sağlık ve Sosyal Refah Araştırmaları Dergisi. (2023) 5:36–53.

[44] Demir V Demir A. Sanatla Terapi Programı ve Etkileşim Grubu Uygulamasının Ruhsal Belirti Düzeyleri Üzerindeki Etkisi. Türkiye Bütüncül Psikoterapi Dergisi. (2019) 1:97–120. doi: 10.55050/sarad.1182479

